# Readmissions to a Surgical Intensive Care Unit: Incidence and Risk Stratification for Personalized Patient Care

**DOI:** 10.3390/jpm15120618

**Published:** 2025-12-11

**Authors:** Silvia Ramos, Rafael Ramos Fernández, Raul Sevilla, Eneko Cabezuelo, Alberto Calvo, Raquel Vela, Claudia Menendez, Sergio Garcia Ramos, Javier Hortal Iglesias, Ignacio Garutti, Patricia Piñeiro

**Affiliations:** 1 Department of Anesthesiology and Critical Care, Gregorio Marañón University Hospital, 28007 Madrid, Spain; 2 Instituto de Investigación Sanitaria Gregorio Marañón (ISGM), 28007 Madrid, Spain; 3 School of Medicine, Universidad Complutense de Madrid, 28040 Marid, Spain

**Keywords:** patient readmission, surgical ICU, mortality rate

## Abstract

**Background/Objectives**: Unplanned readmission to the surgical intensive care unit (UR-SICU) is a serious adverse event linked to higher morbidity, prolonged stay, and increased mortality. Most evidence derives from mixed ICUs, limiting applicability to surgical cohorts. We aimed to identify risk factors for UR-SICU and assess their impact on outcomes. **Methods**: We performed a retrospective cohort study of adults admitted to a 20-bed SICU in a tertiary hospital between June 2021 and December 2022 after non-cardiac surgery (elective, urgent, trauma, or liver transplantation). Patients dying during the first SICU stay or transferred to another ICU were excluded. Demographics, comorbidities, severity scores, treatments, and complications were recorded. Logistic regression identified predictors. Kaplan–Meier curves analyzed survival. **Results**: Among 1361 patients, 82 (6.4%) required UR-SICU. Half were surgical (mainly hemorrhage and sepsis), while respiratory and infectious complications predominated among medical readmissions. Independent predictors for UR-SICU were age (OR 1.03/year; *p* = 0.002), active malignancy (OR 1.79; *p* = 0.012), and delirium during the first SICU stay (OR 1.86; *p* = 0.030). UR-SICU patients had longer hospital stays [46 vs. 13 days; *p* < 0.001] and higher hospital mortality (27.1% vs. 1.48%; OR 24.68; *p* < 0.001). Mortality remained higher at 6 months (33.3% vs. 7.1%) and 1 year (42.3% vs. 11.1%). **Conclusions**: UR-SICU occurred in 6.4% of patients and was independently associated with age, malignancy, and delirium. Readmission was strongly linked to prolonged hospitalization and increased short- and long-term mortality. Early recognition of high-risk patients and targeted, personalized preventive strategies may help reduce avoidable readmissions.

## 1. Introduction

Timely discharge of patients from intensive care units (ICUs) is essential to optimize resources, avoid unnecessary stays, and ensure the availability of beds for new patients requiring critical care [1,2]. Premature discharge may imply transferring the patient to a care setting with an insufficient level of attention, which increases the risk of clinical deterioration and readmission to the ICU. On the other hand, even when discharge occurs at the appropriate time, patients may develop complications during their hospital stay that make it necessary for them to be readmitted to the ICU, a circumstance that is often difficult to predict. In any case, the need for readmission leads to greater morbidity and mortality, prolonged hospital stays, and increased associated costs [3].

Much of the available evidence on ICU readmission rates comes from studies conducted in mixed ICUs, which represents a limitation when extrapolating the results to units with a specific patient case mix [4]. Medical ICU (MICU) and surgical ICU (SICU) present substantial differences across multiple dimensions, including mortality rates [5], level of intervention [6], presence of comorbidities [7,8,9], and baseline functional and nutritional status [10], as well as the incidence of treatment-related adverse events [11].

Unplanned readmission to the ICU (UR-ICU) not only represents an adverse event with a potential negative impact on the patient’s prognosis but has also been proposed as a quality-of-care indicator [3,12].

UR-ICU rates vary widely, ranging from 0.9% to 16% [3,13,14,15,16], with associated mortality between 12% and 58% [2,3,14]. This variability may be attributed to multiple factors, such as the type of ICU (medical, surgical, or mixed) [6,11,17] differences in discharge criteria across units; the availability of resources, such as step-down units [16,18]; and patient clinical characteristics [14,17,19,20,21].

Identifying the factors associated with these readmissions is essential to designing individualized preventive strategies, improving discharge processes, and ensuring a personalized, safer, and more efficient continuity of care.

The primary objective of our study is to analyze risk factors for UR-ICU in a SICU (UR-SICU). Secondarily, we aim to analyze whether UR-ICU is associated with increased hospital and long-term mortality

## 2. Materials and Methods

This study was approved by the Research Ethics Committee and Medicines (CEIm) of Hospital General Universitario Gregorio Marañón (approval code: RE-UCCQ, approval date: 8 September 2025)

This is a retrospective cohort study in a 20-bed SICU of a tertiary hospital.

### 2.1. Inclusion Criteria

All adult patients aged >18 years admitted to the SICU after non-cardiac surgery (urgent or elective, including liver transplantation), as well as polytrauma patients, were included. The study period was 18 months, between 30 June 2021 and 31 December 2022. The at-risk cohort consisted of patients transferred to the ward after their first SICU admission.

### 2.2. Exclusion Criteria

Patients who died during the first SICU admission and those who were transferred to another critical care unit were excluded.

### 2.3. Data and Variables

UR-SICU was defined as the unexpected, unplanned return of the patient to the SICU after discharge from the unit at any time during the same hospitalization. Only the first readmission was considered for analysis in cases with multiple readmissions.

The collected data included demographic variables, previous comorbidities, reason for the primary SICU admission (elective surgical patient, urgent surgical patient, polytrauma, or liver transplantation), severity of illness (Sequential Organ Failure Assessment (SOFA) and Acute Physiology and Chronic Health disease Classification System II (APACHE II), calculated within the first 24 h of admission), duration of organ support (days of vasopressor use and days of mechanical ventilation), complications during the SICU stay (renal failure and need for renal replacement therapy (RRT), days of invasive mechanical ventilation (IMV), need for tracheostomy, pneumonia, bacteremia, infection or colonization by multidrug-resistant organisms (MDRB), delirium, ICU-acquired weakness (ICUAW)), day of discharge (weekday or weekend/holiday), length of the primary SICU stay, hospital length of stay after the primary SICU discharge, and mortality. Subsequently, all patients discharged from the SICU were classified into two groups depending on whether they required an unplanned readmission (UR-SICU) or not (NR-SICU).

In patients who were readmitted, the cause was recorded and classified as medical or surgical depending on whether the complication prompting readmission required a surgical reintervention. Medical causes were categorized according to the primarily affected organ system, distinguishing between cardiologic (cardiac arrest, cardiogenic shock, arrhythmia, and heart failure), respiratory (respiratory failure requiring at least high-flow nasal cannula support and acute respiratory distress syndrome), neurologic (coma, stupor, agitation, and delirium), infectious (sepsis or septic shock of any origin), metabolic (acid–base and electrolyte disturbances and glycemic disorders), or other. Surgical readmissions were subclassified according to the type of complication requiring reintervention, including hemorrhage, sepsis, or septic shock (anastomotic dehiscence, infected collections, bowel obstruction, and intestinal ischemia); decreased level of consciousness secondary to hemorrhagic or infectious complications or development of edema in the context of neurosurgery; and arterial thrombosis, particularly in patients undergoing vascular surgery or liver transplantation.

Patient characteristics and outcomes were compared between those with and without readmission.

Hospital mortality included all patients who died either in the SICU after readmission or during hospitalization in the ward.

### 2.4. Organization of the SICU

Our SICU is a 20-bed unit within a tertiary hospital, dedicated to the care of the most complex surgical patients. It admits patients undergoing scheduled non-cardiac surgery who, due to the nature of the procedure, require continuous postoperative monitoring. These include neurosurgery, major vascular surgery, major maxillofacial surgery with microvascular reconstruction, esophagectomy, pancreaticoduodenectomy, total pancreatectomy, major hepatic resection, extensive abdominal cytoreduction with or without hyperthermic intraperitoneal chemotherapy, pneumonectomy, liver transplantation, and, in general, any long-duration surgery (>8 h). It also admits patients who, after non-cardiac surgery, require organ support due to intraoperative adverse events or high perioperative risk.

Postoperative patients of intermediate complexity, who in other centers would be admitted to the ICU, are admitted to our hospital to the post-anesthesia recovery unit, which has continuous 24 h medical coverage. If any of these patients experience clinical deterioration requiring life support, they are transferred to the SICU.

The unit also cares for polytrauma patients with an Injury Severity Score (ISS) greater than 15, with or without traumatic brain injury.

Similarly, patients who, during their hospitalization after surgery or polytrauma, initially do not require critical care but develop medical–surgical complications that make life support necessary, are admitted to the SICU.

Medical care is provided by a closed team of anesthesiologists with specific training in critical care, with continuous on-site presence of at least one physician, organized in 8 h shifts and 24 h on-call duties, and supported by resident physicians. Clinical management is carried out in a multidisciplinary fashion, with daily participation of different surgical teams, nursing staff, and other support services, such as nephrology, clinical microbiology, rehabilitation, and physiotherapy. Decisions regarding admission, readmission, and discharge are the responsibility of the SICU medical team.

Patients are transferred to the ward when they no longer require continuous monitoring, are hemodynamically stable, require oxygen therapy only via nasal cannula, are not dependent on continuous renal replacement therapy, and have an acceptable general condition. If a tracheostomy was performed during the SICU stay due to prolonged intubation or neuromuscular disease of the critically ill patient, transfer to the ward takes place once decannulation has been achieved. If the patient requires a prolonged SICU stay, transfer to the ward is usually performed from Monday to Thursday.

### 2.5. Statistical Analysis

As we have not previous incidence of readmission in our mixed ICU, we performed a pilot study during 18 months study period. Continuous variables were expressed as median and interquartile range, and ordinal/categorical variables were expressed as number and percentage of the total.

Nonparametric tests were used, such as the Mann–Whitney test for comparison of continuous variables between patients with UR-SICU and those without and the chi-square test or Fisher’s exact test for categorical variables. A multivariate analysis was performed with the variables that showed *p* < 0.1 in those comparisons in order to identify factors associated with readmission.

SPSS software (version 21.0) was used for statistical analysis.

Finally, Kaplan–Meier curves were generated to compare hospital survival in patients in the UR-SICU and NR-SICU groups. In addition, another comparison of survival was made between patients who had been discharged from the hospital.

## 3. Results

**Figure 1:**
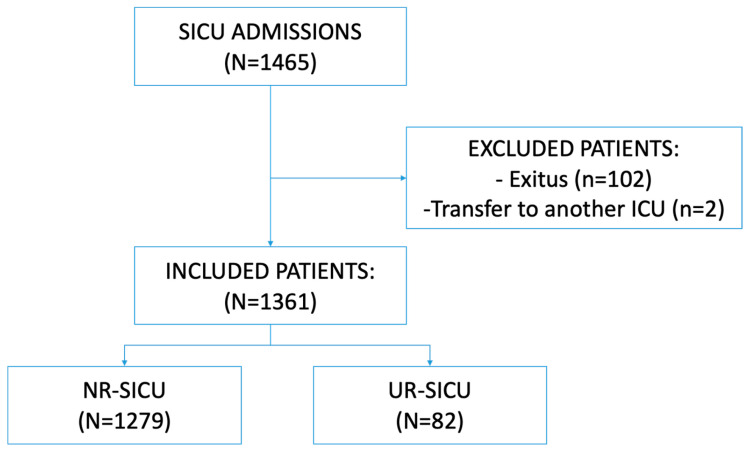
Patient flow. SICU: surgical intensive care unit. NR-SICU: no readmission to the SICU. UR-SICU: unplanned readmission to the SICU.

A total of 1465 patients were initially included in the study. Subsequently, 104 were excluded because they either died during their first SICU stay (*n* = 102) or were transferred to another ICU before discharge to the ward (*n* = 2). Of the 1361 patients ultimately included in the analysis, 82 (6.4%) were readmitted to the SICU after having been transferred to the ward (Figure 1). In total, 25 patients were readmitted within 48 h of discharge (1.6% of all discharges), 13 between days 3 and 7, and the remaining 44 after the first week post-discharge.

### 3.1. Characteristics of Readmissions

Of the 82 patients in the UR-SICU group, 41 (50%) were readmitted for a surgical reason. In 36 (72%) patients, reintervention was due to hemorrhage or sepsis related to a complication of the initial surgery. When the reason for readmission was medical, respiratory and infectious causes predominated, together accounting for 21 cases (42%) (Table 1).

### 3.2. Risk Factors for UR-SICU

Regarding patient characteristics at the first SICU admission (Table 2), patients in the UR-SICU group were older and had higher APACHE II and SOFA severity scores. With respect to comorbidities, readmission was associated with a higher prevalence of active neoplasia, diabetes mellitus, and arterial hypertension. The reason for the first admission also differed, with initial admission for a medical complication in surgical patients being more frequent among those who were readmitted.

Regarding treatments and complications during the SICU stay (Table 3), patients in the UR-SICU group more frequently experienced renal failure and delirium and more often received vasoactive drugs and renal replacement therapy (RRT). Likewise, their initial SICU stay was longer. No statistically significant differences were found in the duration of invasive mechanical ventilation or in the incidence of pneumonia, bacteremia, or infections caused by multidrug-resistant microorganisms

To identify independent predictors of SICU readmission, a logistic regression analysis (Table 4) was performed, including the following variables: age, presence of active neoplasia, delirium, and tracheostomy. The model was statistically significant (χ^2^ = 28.187; *p* < 0.001). Age was significantly associated with a higher risk of readmission, as were the presence of active neoplasia and the development of delirium. Tracheostomy also showed a trend toward being a predictor of readmission but did not reach statistical significance.

### 3.3. Impact of Readmission

Regarding clinical outcomes, patients in the UR-SICU group had longer hospital stays [46 (23–72) vs. 13 (7–24) days; *p* < 0.001]. A total of 10.97% of patients who were readmitted died during their second SICU stay. Overall hospital mortality (death during SICU readmission or after transfer to the ward) was significantly higher in the readmission group (27.1% vs. 1.48%; *p* < 0.001; odds ratio: 24.68 (95% CI 12.67–48.10), *p* < 0.001). At six months (33.3% vs. 7.13%; *p* < 0.001; odds ratio: 6.51 (95% CI 3.88–10.91), *p* < 0.001) and at one year (42.3% vs. 11.12%; *p* < 0.001; odds ratio: 5.85 (95% CI 3.61–9.48), *p* < 0.001), mortality differences between groups persisted.

**Figure 2:**
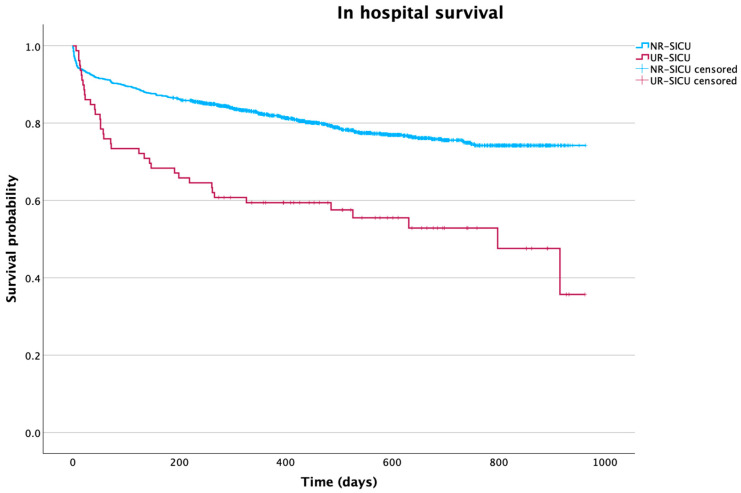
Kaplan–Meier curve showing differences in survival between groups over time after transfer to the ward. NR-SICU: no readmission to the SICU; UR-SICU: unplanned readmission to the SICU.

Survival analysis using Kaplan–Meier curves showed differences in survival between the two groups. Analyzing survival after SICU discharge following readmission (Figure 2), a significant reduction in survival was observed in patients in the UR-SICU group, with a statistically significant log-rank test (χ^2^ = 68.5; *p* < 0.001). Mean survival after the second ICU discharge was 566.3 days (95% CI: 474.2–658.5).

**Figure 3:**
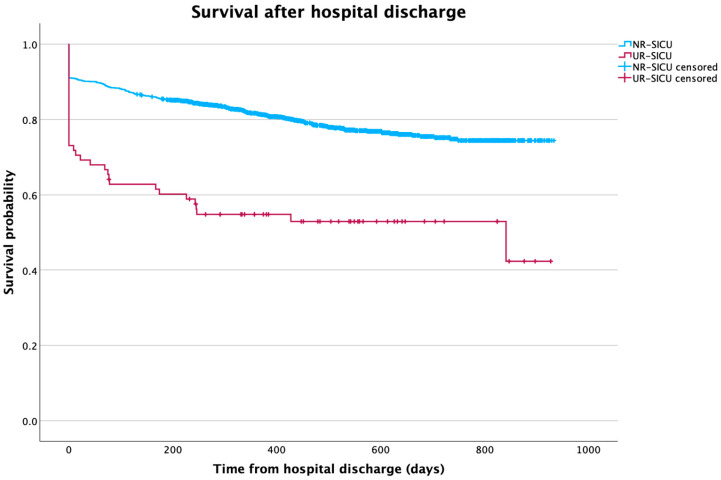
Kaplan–Meier curve showing differences in survival between groups over time after hospital discharge. NR-SICU: no readmission to the SICU; UR-SICU: unplanned readmission to the SICU.

When analyzing survival after hospital discharge (Figure 3), differences were also observed. Patients in the UR-ICU group had significantly lower survival compared with those who were not readmitted (log-rank: χ^2^ = 70.7; *p* < 0.001). In this group, mean survival was 524.3 days (95% CI: 424.6–624.1), whereas patients without readmission reached a mean of 808.6 days (95% CI: 729.3–824.9).

## 4. Discussion

In our cohort of surgical patients, 6.4% of those discharged to the ward required readmission to the SICU during hospitalization. Patients in the UR-SICU group were older and more severely ill at their initial admission and had a higher prevalence of active neoplasia, diabetes, and arterial hypertension compared with those who were not readmitted. Likewise, those who, during the first admission, received vasoactive drugs or renal replacement therapy or developed delirium showed a higher likelihood of readmission. Patients in the UR-SICU group had 27.1 times higher odds of mortality than those in the NUR-SICU group, as well as lower survival after hospital discharge. Age, the presence of neoplasia, and delirium were independently associated with the risk of SICU readmission.

The readmission rate observed in our study is consistent with that reported in previous research, falling at the lower end of the ranges most frequently described in the literature for mixed units, as reported by Rosenberg and Watts in their systematic review (5–14%) [13] or Elliot (0.89–19%) [22]. Regarding SICUs, the rates described tend to be somewhat lower (0.89–13.4%) [14,16], and our cohort also falls within that range.

The interpretation of readmission figures in the literature is complex due to the existence of multiple confounding factors that contribute to the wide dispersion of results [18]. Among these are the heterogeneity of patient case mix and the complexity and organization of hospitals (including the availability of step-down units), as well as variability in the criteria used to select patients considered at risk of readmission [16,18,23]. This methodological diversity limits direct comparability between studies.

In our analysis, patients who died during the first admission and those transferred to other ICUs were excluded. Unlike other authors, we included patients who underwent elective surgery without complications, confirming that their readmission rate was similar to that of patients with urgent admissions. The explanation likely lies in the high complexity of patients treated in our SICU (APACHE II 6–16). In addition, the availability in our center of a post-anesthesia recovery unit where postoperative patients of intermediate complexity are admitted may explain differences with other hospitals, since in other centers, these patients would be admitted to the ICU and would probably show lower readmission rates.

Readmission rates are used as an indicator of quality of care, although not without controversy [12,24,25]. While readmission clearly represents a setback in the patient’s recovery trajectory, the usefulness of readmission rates as a measure of ICU or hospital quality is more debatable [26]. There are multiple problems with using it as a quality indicator. The causes of readmissions are not always avoidable, and hospitals with greater complexity in their patients and procedures may present higher rates, without this necessarily implying poorer quality of care [18,24,26]. Nevertheless, given the increase in morbidity, mortality, and associated costs, readmission continues to be a key indicator in the evaluation of ICU quality, especially for the individual monitoring of each ICU within its respective hospital [27,28].

In line with other SICU readmission cohorts, approximately half of readmissions occur after an urgent surgical reintervention, mainly due to bleeding or sepsis [16,29]. In our cohort, when the reason for readmission was medical, respiratory causes predominated [16,29], similar to what occurs in mixed ICUs [13,14,23,30].

In our cohort, we identified several factors associated with a higher risk of SICU readmission, several of which have already been described in the literature, such as age, severity at admission, the use of vasoactive drugs, the need for renal replacement therapy, and the presence of cardiovascular risk factors such as diabetes and arterial hypertension [2,13,15,16,17,21,22,31].

Transfer to the ward during weekends or days with reduced medical presence has been reported as a risk factor for readmission, although inconsistently [4,16,22]. In our center, it is customary not to transfer patients with prolonged SICU stays to the ward during weekends in order to ensure adequate handover of information to the responsible medical team. The quality of information transfer has been highlighted as one of the main measures to prevent avoidable readmissions [1,15,28].

In the multivariate analysis, older age, the presence of active neoplasia, and the development of delirium during the initial ICU admission were independent predictors of UR-SICU. The association between age and higher risk of readmission (OR = 1.026 per year) is consistent with previous studies that recognize age as a marker of lower physiological reserve, greater susceptibility to organ dysfunction, and poorer tolerance to complications [16,32,33]. The presence of active malignancy nearly doubled the risk of readmission (OR = 1.79), a finding consistent with studies describing readmission rates of up to 20% in oncological patients [34]. Active malignant disease leads to a higher incidence of complications, immunosuppression, malnutrition, and metabolic disturbances [31,34], all of which contribute to greater frailty. In addition, it has been identified as a predictor of hospital mortality and of mortality after ICU readmission, even after adjusting for initial severity [30,35].

The development of delirium during the initial SICU stay was confirmed as an independent predictor of UR-SICU (OR = 1.86), in contrast with some previous studies [17]. The prevalence of delirium as a risk factor for readmission has been scarcely reported in large studies, probably due to difficulties in its proper definition and recording. Delirium, understood as an acute neurological dysfunction, reflects a state of systemic stress characterized by neuroinflammation, alterations of the blood–brain barrier, and neurotransmitter imbalance [36,37]. It has a multifactorial etiology, linked to patient severity but with potentially modifiable factors, making it a relevant target for intervention [38]. Its presence is associated not only with worse immediate prognosis but also with higher short- and long-term mortality and persistent cognitive impairment [38]. Reducing its incidence has become one of the main care objectives in ICUs, with the systematic implementation of campaigns and strategies aimed at optimizing its detection, characterization, and management [37,39,40,41,42].

The performance of tracheostomy showed a trend toward an independent association with the risk of UR-SICU, although it did not reach statistical significance in the multivariate analysis. This procedure is usually indicated in the context of respiratory weaning in patients with ICUAW and prolonged intubation [43]. In our unit, the systematic implementation of physiotherapy and rehabilitation programs from the early phase of tracheostomy until decannulation, following expert recommendations [43,44], may have contributed to mitigating the associated frailty in these patients.

In our cohort, the UR-SICU group had a longer hospital stay with a significant increase in hospital mortality, with an odds of mortality of 24.68 (27.1% vs. 1.48%) compared with NUR-SICU. Previous studies have reported mortality rates between 12% and 58% and a 4- to 11-fold higher risk for patients who are readmitted compared with those who are not [3,14,22]. Our mortality results in the UR-SICU group are consistent with these figures; however, the mortality observed in NUR-SICU (1.48%) is lower than that described in the literature (3–9.1%) [16,29]. This difference could be explained by the inclusion in our study of patients undergoing elective surgery. Although these patients had a readmission frequency similar to that of urgent surgeries (46.9% vs. 41.5%), the combination of a low overall readmission rate of 6.4% in our cohort, together with the better baseline condition and preoperative optimization of elective patients, may have influenced the mortality figures. Our cohort of patients presents a high prevalence of active oncologic disease, significantly higher in the NR-SICU group, around 45%, greater than that reported in previous studies (5–22%) [14,16,17]. This does not appear to have had a major impact on in-hospital mortality figures but is probably influencing medium- and long-term mortality after hospital discharge due to the added frailty of these patients [45].

In our cohort, the UR-SICU group showed reduced survival after hospital discharge. This is consistent with the concept of post-hospital syndrome proposed by Krumholz [45], according to which patients present a transient vulnerability after hospital discharge, promoted by physical and cognitive disability, malnutrition, sleep deprivation, and persistence of delirium. Critically ill patients who survive an ICU admission are especially vulnerable [5,46], and readmission appears to accentuate this risk, as suggested by our results.

In summary, our findings reinforce the well-known negative clinical impact of ICU readmission, not only in terms of prolonged length of stay and increased number of complications but also in terms of the reduction in medium- and long-term survival. Therefore, it is essential to identify early those patients at higher risk of readmission and to implement individualized strategies targeting modifiable factors in order to optimize post-discharge care and reduce preventable readmissions.

Among the main strengths of this study are the cohort size and the homogeneity of patient characteristics, which provide a valuable perspective on readmission in the context of an SICU.

Nevertheless, there are limitations to consider. The retrospective nature of the study prevented the collection of relevant information, such as the clinical appropriateness of readmission, patients discharged with a do-not-readmit order, those who required readmission to another ICU in our hospital, or those who were transferred to another center before hospital discharge—factors that could substantially modify the number and characteristics of our at-risk cohort. In addition, the multivariate analysis may have been influenced by unmeasured variables that affected the results.

## 5. Conclusions

UR-SICU was associated with an odds of mortality of 24 during hospitalization and an odds of mortality of 6 at one year. Age, the presence of neoplasia, and delirium were independently associated with the risk of readmission. Recognizing these factors in high-risk patients is critical to designing individualized preventive strategies, optimizing care after ICU discharge, and advancing toward personalized medicine in critical care.

## Figures and Tables

**Table 1:** Reasons for readmission.

Readmission Due to Medical Cause	41 (50%)	Readmission Due to Surgical Cause	41 (50%)
Cardiologic	6 (7.3%)	Hemorrhage	19 (23.2%)
Respiratory	11 (13.4%)	Sepsis or septic shock	17 (20.7%)
Neurologic	7 (8.5%)	Decreased level of consciousness	2 (2.4%)
Infectious	10 (12.2%)	Arterial thrombosis	3 (3.7%)
Metabolic	4 (4.9%)		
Other	3 (3.7%)		

**Table 2:** Patient characteristics at primary SICU admission.

Variable	NR-SICU (*n* = 1279)	UR-SICU (*n* = 82)	*p* Value
Age ^a^	60 (48–71)	66 (56–73)	<0.001
Sex. Male ^b^	749 (58.6%)	50 (61%)	0.667
**Severity scale ^a^**			
APACHE II	9 (6–13)	13 (9–16)	<0.001
SOFA	2 (1–5)	4 (1–6)	0.002
**Comorbidities ^b^**			
Neoplasia	471 (36.8%)	43 (52.4%)	0.005
Ischemic heart disease	105 (8.2%)	7 (8.5%)	0.917
Heart failure	67 (5.2%)	7 (8.5%)	0.202
Liver disease	123 (9.9%)	10 (12.2%)	0.446
CKD	97 (7.6%)	9 (11.0%)	0.267
COPD	162 (12.7%)	14 (17.1%)	0.249
Diabetes	242 (18.9%)	23 (28.0%)	0.043
Hypertension	534 (41.8%)	45 (54.9%)	0.020
**Primary diagnosis for SICU admission ^b^**			<0.001
Postoperative care. Planned admission.	678 (53.0%)	49 (59.7%)	
Medical complication in a postoperative patient	90 (7%)	11 (13.4%)	
Trauma	207 (16.2%)	1 (2.4%)	
Emergency surgery	228 (17.8%)	19 (23.2%)	
Liver transplant	76 (5.9%)	2 (2.4%)	
**Nature of first SICU admission ^b^**			
Emergency admission	601 (46.9%)	34 (41.5%)	0.331

^a^ median (interquartile range). ^b^ number (%). NR-SICU: no readmission to the SICU. UR-SICU: unplanned readmission to the SICU. CKD: chronic kidney disease. COPD: chronic obstructive pulmonary disease.

**Table 3:** Characteristics, treatments, and complications during the primary SICU stay.

Variable	NR-SICU (*n* = 1279)	UR-SICU (*n* = 82)	*p* Value	Univariate OR, *p* Value
Vasoactive drugs ^b^	359 (30.6%)	31 (42.5%)	0.034	OR 1.67 (IC95% 1.03–2.70), *p* = 0.03
Days of invasive ventilation ^a^	6 (4–9)	6 (4–10)	0.303	N/A
Tracheostomy ^b^	111 (8.6%)	12 (14.6%)	0.068	OR 1.80 (IC95% 0.94–3.43), *p* = 0.07
ARDS ^b^	19 (1.5%)	2 (2.4%)	0.497	OR 1.65 (IC95% 0.38–7.24), *p* = 0.50
Pneumonia ^b^	92 (7.2%)	10 (12.2%)	0.095	OR 1.79 (IC95% 0.89–3.58), *p* = 0.10
Bacteremia ^b^	32 (2.5%)	4 (5.2%)	0.158	OR 2.10 (IC95% 0.73–6.15), *p* = 0.16
Infection or colonization by MDRB ^b^	67 (5.3%)	6 (7.8%)	0.346	OR 1.51 (IC95% 0.63–3.61), *p* = 0.34
Renal insufficiency ^b^	264 (20.7%)	26 (31.7%)	0.025	OR 1.78 (IC95% 1.09–2.89), *p* = 0.02
RRT	49 (3.8%)	10 (12.2%)	<0.001	OR 3.48 (IC95% 1.69–7.16), *p* < 0.001
Delirium	148 (11.6%)	19 (23.2%)	0.002	OR 2.30 (IC95% 1.34–3.95), *p* = 0.002
ICUAW	67 (5.2%)	6 (7.3%)	0.441	OR 1.42 (IC95% 0.60–3.39), *p* = 0.42
Days of primary SICU admission ^a^	2 (1–5)	3 (2–8)	0.00	N/A
Transfer day. Weekday ^b^	1030 (80.5%)	70 (85.36%)	0.281	OR 1.41 (IC95% 0.75–2.64), *p* = 0.283

^a^ median (interquartile range). ^b^ number (%). NR-SICU: no readmission to the SICU; UR-SICU: unplanned readmission to the SICU ARDS: acute respiratory distress syndrome; MDRB: multidrug-resistant bacteria; RRT: renal replacement therapy; ICUAW: ICU-acquired weakness. N/A: not applicable.

**Table 4:** Logistic regression analysis.

Variable	Odds Ratio (OR)	IC 95%	*p* Value
Age	1.026	1.010–1.042	0.002
Neoplasia	1.792	1.140–2.818	0.012
Delirium	1.855	1.061–3.243	0.030
Tracheostomy	1.638	0.850–3.156	0.140

## Data Availability

The original contributions presented in this study are included in the article. Further inquiries can be directed to the corresponding author.

## Abbreviations

The following abbreviations are used in this manuscript:

ICU	Intensive care unit
MICU	Medical intensive care unit
SICU	Surgical intensive care unit
UR-SICU	Unplanned readmission to the surgical intensive care unit
UR-ICU	Unplanned readmission to the intensive care unit
NR-ICU	No readmission to the intensive care unit
APACHE	Acute Physiology and Chronic Health Disease Classification System II
SOFA	Sequential Organ Failure Assessment
RRT	Renal replacement therapy
MDRB	Multidrug-resistant bacteria
ICUAW	ICU-acquired weakness
ISS	Injury severity score
